# Icariin Protects Hippocampal Neurons From Endoplasmic Reticulum Stress and NF-κB Mediated Apoptosis in Fetal Rat Hippocampal Neurons and Asthma Rats

**DOI:** 10.3389/fphar.2019.01660

**Published:** 2020-01-31

**Authors:** Jiaqi Liu, Lumei Liu, Jing Sun, Qingli Luo, Chen Yan, Hongying Zhang, Feng Liu, Ying Wei, Jingcheng Dong

**Affiliations:** ^1^ Department of Integrative Medicine, Huashan Hospital, Fudan University, Shanghai, China; ^2^ Institutes of Integrative Medicine, Fudan University, Shanghai, China

**Keywords:** asthma, icariin, endoplasmic reticulum stress, hippocampal neurons, apoptosis, NF-κB

## Abstract

Icariin is a main component of the Chinese medicinal plant *Epimedium brevicornu* Maxim, exhibits potent activity against inflammatory diseases. Our previous data demonstrated the valid bioactivity of icariin on mitigating rodent asthma. Endoplasmic reticulum (ER) stress and nuclear factor-κB (NF-κB) pathway were involved in the pathogenesis of asthma. However, it remains poorly defined that whether icariin could inhibit ER stress and NF-κB mediated apoptosis in asthma and further influence the central neural system. Herein, we investigated the effects of icariin on primary cultured fetal rat hippocampal neurons and OVA_LPS_-OVA induced asthma rat model. Asthma rat models were established by ovalbumin (OVA) and lipopolysaccharide (LPS) intraperitoneal injection and OVA inhalational challenge. Airway resistance was analyzed to evaluate lung function after last challenge and pathological changes were detected on lung tissues. Assessment of inflammatory cells counts in bronchoalveolar lavage fluids (BALF) were performed and ELISA was used to determine levels of interleukin (IL)-1β, tumor necrosis factor-α, IL-6, and interferon-γ in serum. Protein expression of BiP and IRE-1α, XBP-1s and phosphorylation-IκBα (p-IκBα), IκBα, and p65 as well as cytochrome c, caspase-3 (cleaved caspase-3), and caspase-9 (cleaved caspase-9) were tested by Western blot. We found that icariin could remarkably improve pulmonary function and reduce inflammatory cells in the lung, levels of inflammatory cytokines, and ER stress related proteins as well as NF-κB were prominently suppressed by icariin. Our results suggested that icariin had an inhibitory effect on airway inflammation and neuroprotective effect on ER stress and NF-κB mediated apoptosis in asthma rats and cultured fetal rat hippocampal neurons, which may provide new mechanistic insights into the asthma prevention and treatment of icariin.

## Introduction

Asthma is believed to be a most common respiratory disorder threatening hundreds of millions individuals worldwide, and the prevalence has increased considerably over the past decades. Inflammation of the airway is a key characteristic of asthma, the release of mediators from the inflammatory cells including eosinophils, lymphocytes, mast cells, and neutrophils (Amin, 2016; Januskevicius et al., 2016; Su et al., 2018), as well as inflammatory cytokines, such as interferon (IFN)-γ, interleukin (IL)-1β, IL-6, and tumor necrosis factor (TNF)-α (Yokoyama et al., 1995; Wei-xu et al., 2014; Huang et al., 2016; Diao et al., 2017), has been proposed to contribute directly or indirectly to changes in airway structure and function.

As a defining feature of the stress response (Buske-Kirschbaum, 2009), activation of the hypothalamic-pituitary-adrenal (HPA) axis has been demonstrated to be strongly associated with asthma pathogenesis (Wei et al., 2017), and corticotropin releasing hormone (CRH) that secreted by the hypothalamus plays a central role in this activation. Recent studies suggested that hippocampus was not only a high regulation center of the HPA axis in the limbic system, but also a sensitive area of stress injury, and CRH may lead to stress-induced hippocampal dysfunction (Lyons et al., 1991; Brunson et al., 2001; Ivy et al., 2010). As an inflammatory and stress related disease (Plourde et al., 2017), asthma adults were found significantly smaller volume of hippocampal as compared to those without asthma (Carlson et al., 2017), indicating the close relevant between hippocampal changes and asthma evolution. However, the underlying mechanism has not been well elucidated and an effective treatment is still insufficient.

Endoplasmic reticulum (ER) serves as an organelle and plays a critical role in biosynthesis, rectification of protein folding, and modifications of posttranslational proteins. Loss of ER homeostasis evokes the ER stress response, leading to activation of unfolded protein response (UPR), a pivotal feature of inflammatory diseases (Osorio et al., 2013). BiP (binding immunoglobulin protein), a molecular chaperone, dissociates with IRE1-α (Inositol-requiring kinase 1) in response to ER stress and associates with unfolded proteins in order to maintain the correct structure. Co-related to dissociation of BiP, endoribonuclease domain of IRE-1α is activated by transautophosphorylation. Activated IRE-1α then, by cleaving mRNA of XBP-1 (X-box binding protein 1) and activating its transcription activity triggers UPR (Janssens et al., 2014). UPR signaling protects cells from ER stress through the expansion of amount of ER in the cell, and increases the degradation of misfolded proteins, and decreases the synthesis of new proteins (Zhang et al., 2012). Studies have suggested that protein synthesis and secretion could be affected by excess ER stress, and induces generation of reactive oxygen species, and provokes inflammation by activation of nuclear factor-κB (NF-κB) activation (Kincaid and Cooper, 2007) and mediates cell death through apoptosis with activation of cytochrome c and the downstream caspase-9 and caspase-3. Based on the correlation between ER stress and various inflammatory responses, targeting on ER stress could be promising approaches for bronchial asthma treatment (Kim et al., 2013).

Icariin, extracted and purified from the Chinese medicinal plant Epimedium (Chinese name: Yin Yang Huo) exhibits potent anti-inflammatory activity against inflammatory diseases (Liu et al., 2011; Wei et al., 2015). However, the effects and underlying molecular mechanisms of icariin on ER stress and airway inflammation have not been fully elucidated. In this study, we aim to examine the regulatory effects and the related mechanisms of icariin on ER stress and airway inflammation.

## Materials and Methods

### Drug and Reagents

**Figure 1:**
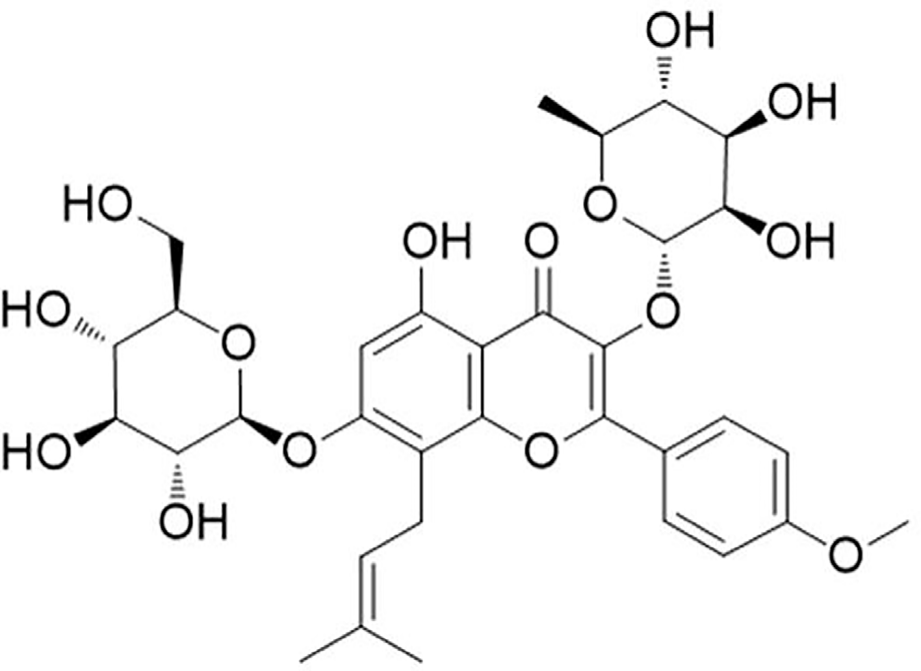
Molecular structure of icariin. [C_33_H_40_O_15_; molecular weight = 676.67 (Shen and Wang, 2018)].

Icariin (purity ≥ 99%), chemical structure shown in **Figure 1**, was provided by Shanghai Winherb Medical Technology Co., Ltd. Cell culture reagents including fetal bovine serum (FBS), DMEM medium, streptomycin, and penicillin were obtained from GIBICO (Grand Island, NY). Lipopolysaccharide (LPS) was from *Escherichia coli* (055:B5), dimethyl sulfoxide (DMSO), ovalbumin (OVA, grade V), aluminum hydroxide, and methacholine (Mch) were supplied by Sigma-Aldrich Co. LLC. Rat ELISA kits of CRH, IFN-γ, IL-1β, IL-6, and TNF-α were obtained from Raybiotech. Anti-rabbit BiP, IRE-1α, p65, p-IκBα, IκBα antibody were purchased from Cell Signaling Technology, Inc. and β-actin and primers were provided by Sangon Biotech (Shanghai, China) Co., Ltd. Anti-rabbit CRH antibody was supplied by Boster (Wuhan, China) Co., Ltd. and CRH peptide was from Chinapeptides (Shanghai, China) Co., Ltd.

### Cell Culture and Treatments

Primary hippocampal neurons were obtained from newborn (less than 24 h) Sprague-Dawley (SD) rats, all procedures were performed following the approval of Fudan University and international guidelines on ethical treatment of experimental animals. Primary hippocampal neurons were prepared as previous description (Liu et al., 2011). In brief, hippocampal tissues were dissected, gently minced, and trypsinized (Typsin 0.25%, 15 min) at room temperature, and the digestion was stopped by DMEM plus 10% FBS. After being filtered, centrifuged, and washed, cells were plated on poly-L-lysine (molecular weight 30,000–70,000, 0.1 mg/ml; Sigma, St. Louis, MO, USA) coated plates or glass coverslips in DMEM containing 10% FBS for 24 h (37 °C, 5% CO_2_) before being changed into neurobasal medium supplemented with B27, 100 U/ml penicillin, and 100 μg/ml streptomycin. The cultured neurons were identified by immunocytochemistry with the antibody against MAP-2, which is a marker for neurons. The culture medium was changed every 2 days. Cells were grown to 80% confluence prior to treatment. Icariin was dissolved in culture-grade DMSO (final concentration < 0.1%) in serum-free media. To investigate the protective effect of icariin, primary cultured rat hippocampal neurons were treated with 10, 20, and 50 nM of icariin for 2 h and then treated with 5 nM CRH for 24 h while the CRH group received 5 nM CRH treatment alone.

### Cell Viability Assays

Primary hippocampal neurons were seeded into 96-well plates and cultured overnight. After various treatments, cell viability was determined by 3-(4,5)-dimethylthiahiazo (-z-y1)-3,5-diphenytetrazoliumromide (MTT) assay. MTT was applied to the cultures at a final concentration of 0.5 mg/ml for 4 h at 37°C. The medium was then aspirated, and 100 μl DMSO was added to solubilize the colored formazan product and incubated on the shaker for 15 min. Then absorbance was measured at 490 nm using a micro-plate reader (Bio Tek, VT, USA). Cell viability (%) was expressed as a percentage relative to the untreated control cells.

### Western Blot Analysis

Primary cultured hippocampal neurons were collected and lung tissues were homogenized, both of which were lysed and extracted for protein expression of BiP, IRE-1α, p-IκBα, IκBα, and p65 as well as the apoptosis-related proteins cytochrome c, caspase-9 and caspase-3 expression by Western blot analysis, asthma rat hippocampus were also collected and prepared for CRH protein level determination. 10% SDS-PAGE was used to separate the total protein after extracted. Then the protein samples were electrophoretically transferred onto PVDF membranes. Whereafter, PVDF membranes were blocked with 5% BSA for 2 h at room temperature and subsequently incubated with primary and secondary antibodies. The immunoblots were incubated with primary antibodies BiP, IRE-1α, p-IκBα, IκBα, and p65 as well as cytochrome c, caspase-9 and caspase-3 (CST 1:1,000) dilution overnight at 4°C, and for secondary antibodies were at 37°C for 2 h with 1:2,000 dilution. At last, the immunoblots were visualized and analyzed with Bio-Rad's Image Lab software (Bio-Rad Laboratories, Inc., Hercules, CA, USA).

### Asthma Model, Grouping, and Treatment

Female specific pathogen-free (SPF) SD rats, 6 to 8 weeks of age and 220–250 g of weight, were purchased from JSJ Laboratory Animal (Shanghai, China) Co., Ltd. and used under a protocol for animal care approved by the Committee on the Ethics of Animal Experiments of Fudan University (Shanghai, China).

We utilized OVA and LPS to duplicate rat asthma models according to literature (Kim et al., 2013). On days 0 and 14, intraperitoneal injection of 1 ml saline containing 100 μg OVA, 30 μg LPS, and 100 mg aluminum hydroxide was implemented for sensitization, from day 21 to day 23, each rat was challenged with 3% OVA nebulization in an individual chamber for 30 min per day utilizing an ultrasonic nebulizer (402AI ultrasonic nebulizer, Yuyue, China).

Rats were randomly divided into five experimental groups, 10 rats per each group, NC: normal control, A: asthma, IC: Icariin (10, 20, 50 mg/kg) groups. NC group rats were sensitized and challenged with equivalent normal saline. IC groups were intragastric administrated of icariin from day 20 to 23 before OVA challenge.

### Measurement of Airway Hyperresponsiveness (AHR)

AHR was determined within 24 h of the last OVA challenge by modular and invasive pulmonary facility (Buxco Electronics Inc., NY) as literature described (Pichavant et al., 2007). Briefly, each rat was anaesthetized before tracheostomized and intubated, gradient of Mch (0.78, 1.56, and 3.12 mg/ml) were nebulized to estimate airway resistance (R_L_) and pulmonary dynamic compliance (Cdyn) and data were shown as a percent change from the baseline value.

### Analysis of Inflammatory Cell in Bronchoalveolar Lavage Fluids (BALF)

Immediately after the determination of AHR, a tracheal tube was inserted and the left lung was lavaged for three times by 0.3 ml aliquots of PBS, then BALF was centrifuged at 800 × g, 4°C for 10 min. The sediments were resuspended in 0.1 ml PBS for cells count in a hemacytometer (HEMAVET 950FS, DREW, USA). The supernatants were collected and stored at −80°C for evaluation of ELISA.

### Pulmonary Histopathological Analysis

The middle lobe of the right lung tissues were detached from the rats and ﬁxed in 4% formalin, embedded in parafﬁn, and thin slices (3–4 μm) from blocks were cut and stained with hematoxylin-eosin (H&E). The histopathological changes were determined by optical microscope (ECLIPSE 80i, Nikon, Japan) to evaluate the inflammatory changes in the lung tissues of different groups of rats. Histopathological evaluation was performed blind on randomized sections. The severity of inflammatory cell infiltration into the lung was assessed by a five-point scoring system (Underwood et al., 1995).

### Serum CRH, IFN-γ, IL-1β, IL-6, and TNF-α Assay

Levels of CRH, IFN-γ, IL-1β, IL-6, and TNF-α in serum were detected by ELISA assay following the manufacturer's instructions.

### Statistical Analysis

Statistic software SPSS 19.0 was used to undertake all analyses. Data was expressed as mean ± SEM. One way analysis of variance (ANOVA) with the LSD test or Dunnett's test were used to perform statistical significance of the differences. P value less than 0.05 was accepted as significant.

## Results

### Icariin Mitigated CRH-Induced Injury in Primary Cultured Rat Hippocampal Neurons

**Figure 2:**
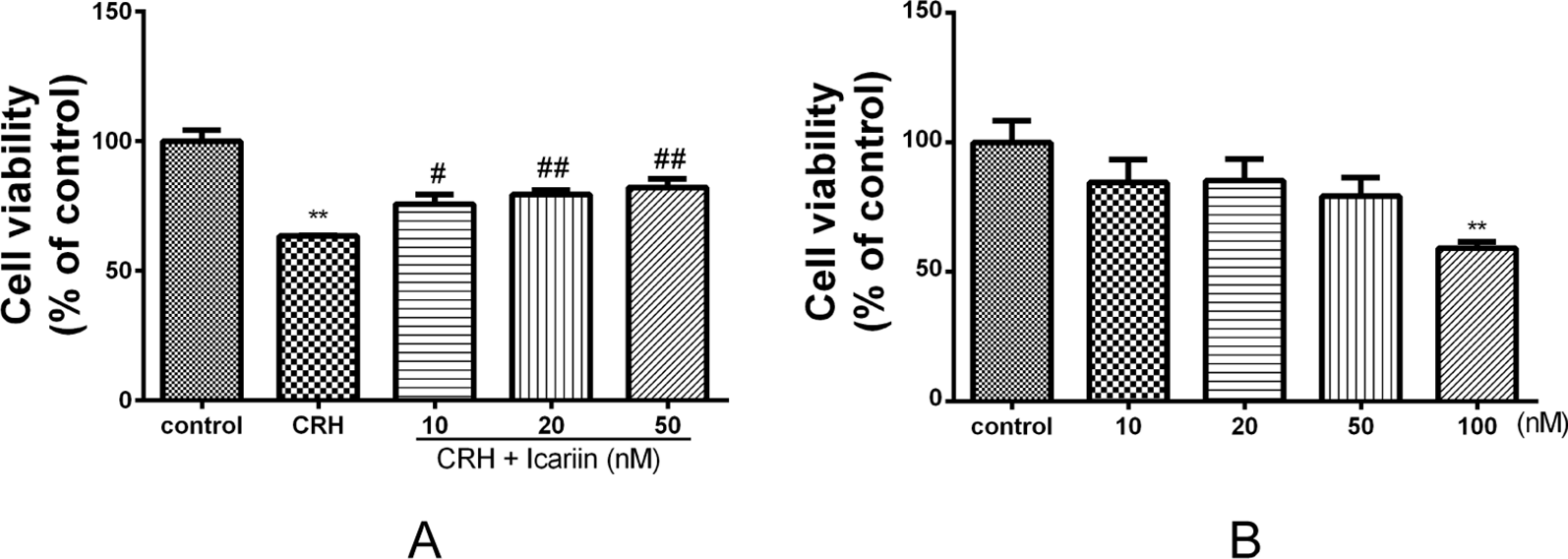
Effects of CRH and icariin on the activity of primary cultured rat hippocampal neurons with MTT assay. Primary cultured rat hippocampal neurons were pretreated with different concentrations of icariin (10, 20, or 50 nM) for 2 h followed by treatment of 5 nM CRH for 24 h **(A).** Primary cultured rat hippocampal neurons were treated with 10–100 nM of icariin, the activity of which were tested with MTT assay **(B).** **p < 0.01 vs. control; ^#^ *p* < 0.05 and ^##^ *p* < 0.01 vs. CRH.

To determine the neurotoxicity of icariin on primary cultured rat hippocampal neurons, cells were treated with 10~100 nM of icariin for 24 h, and few injury was observed in 10~50 nM of icariin (**Figure 2B**), thus we used the three concentrations (10, 20, and 50 nM) in the subsequent experiments. We found that 5 nM of CRH treatment exhibited significant injury to the primary cultured rat hippocampal neurons (*p* < 0.01, **Supplementary Figure 1**). To investigate the effects of icariin on CRH-induced neurotoxicity, primary cultured rat hippocampal neurons were pretreated with different concentrations (10, 20, or 50 nM) or without icariin for 2 h and then incubated with 5 nM CRH for 24 h. MTT assay revealed that icariin promoted neuron viability significantly compared to that in neurons treated with CRH alone (**Figure 2A**).

### Icariin Promoted BiP But Suppressed IRE-1α Protein Expression in Primary Cultured Rat Hippocampal Neurons

**Figure 3:**
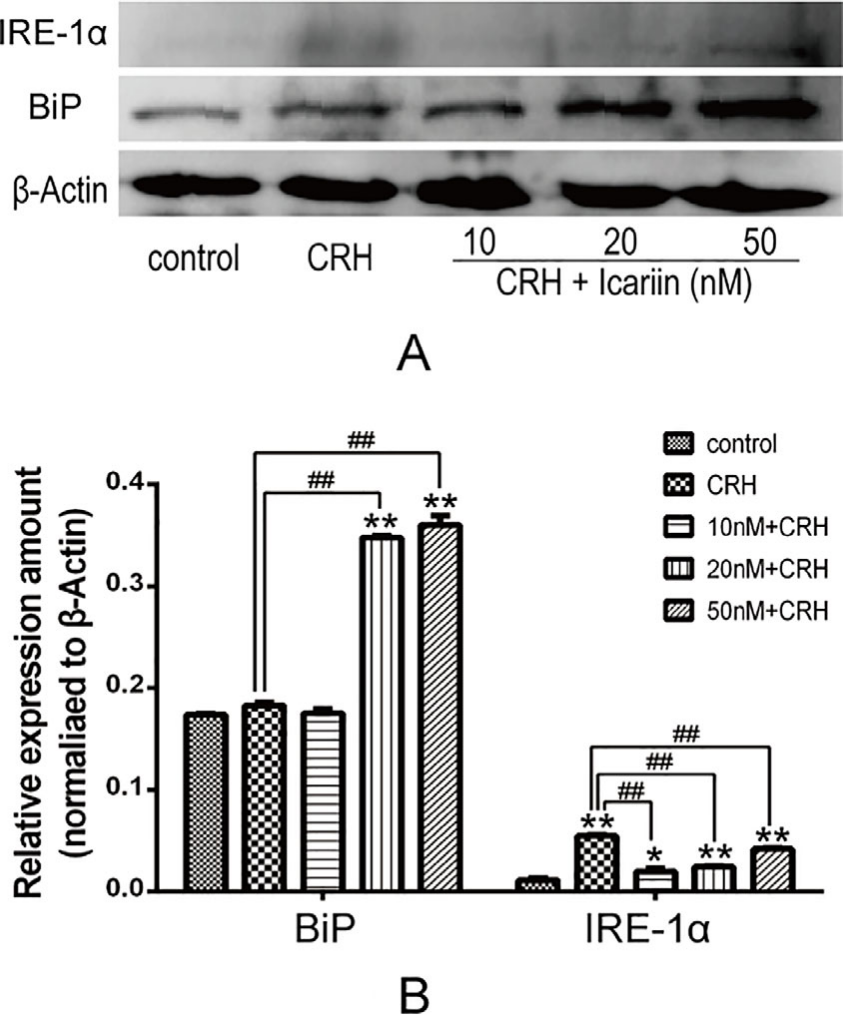
Icariin promoted BiP but suppressed IRE-1α protein expression. Primary cultured rat hippocampal neurons were pretreated with different concentrations of icariin (10, 20 or 50nM) for 2 h followed by treatment of CRH for 24 h. The immunoblotting **(A)** and the corresponding histogram **(B)** showed protein expression in different groups. *p < 0.05 and **p < 0.01 vs control; *^##^p < 0.01* vs CRH.

BiP is an ER stress marker and IRE-1α is a specific UPR signaling component, to investigate whether ER stress is involved in the neuroprotective effect of icariin, BiP, and IRE-1α protein expression in primary cultured rat hippocampal neurons were determined. The results showed that BiP level significantly increased in a dose-dependent manner in primary cultured rat hippocampal neurons pretreated with icariin compared to that treated with CRH alone. IRE-1α protein expression in primary cultured rat hippocampal neurons was markedly augmented by CRH treatment and significantly decreased in primary cultured rat hippocampal neurons after icariin treatment, however, IRE-1α levels among icariin treated groups were not statistically significant (**Figure 3**).

### Icariin Reduced XBP-1s Protein Expression and Inhibited NF-κβ in Primary Cultured Rat Hippocampal Neurons

**Figure 4:**
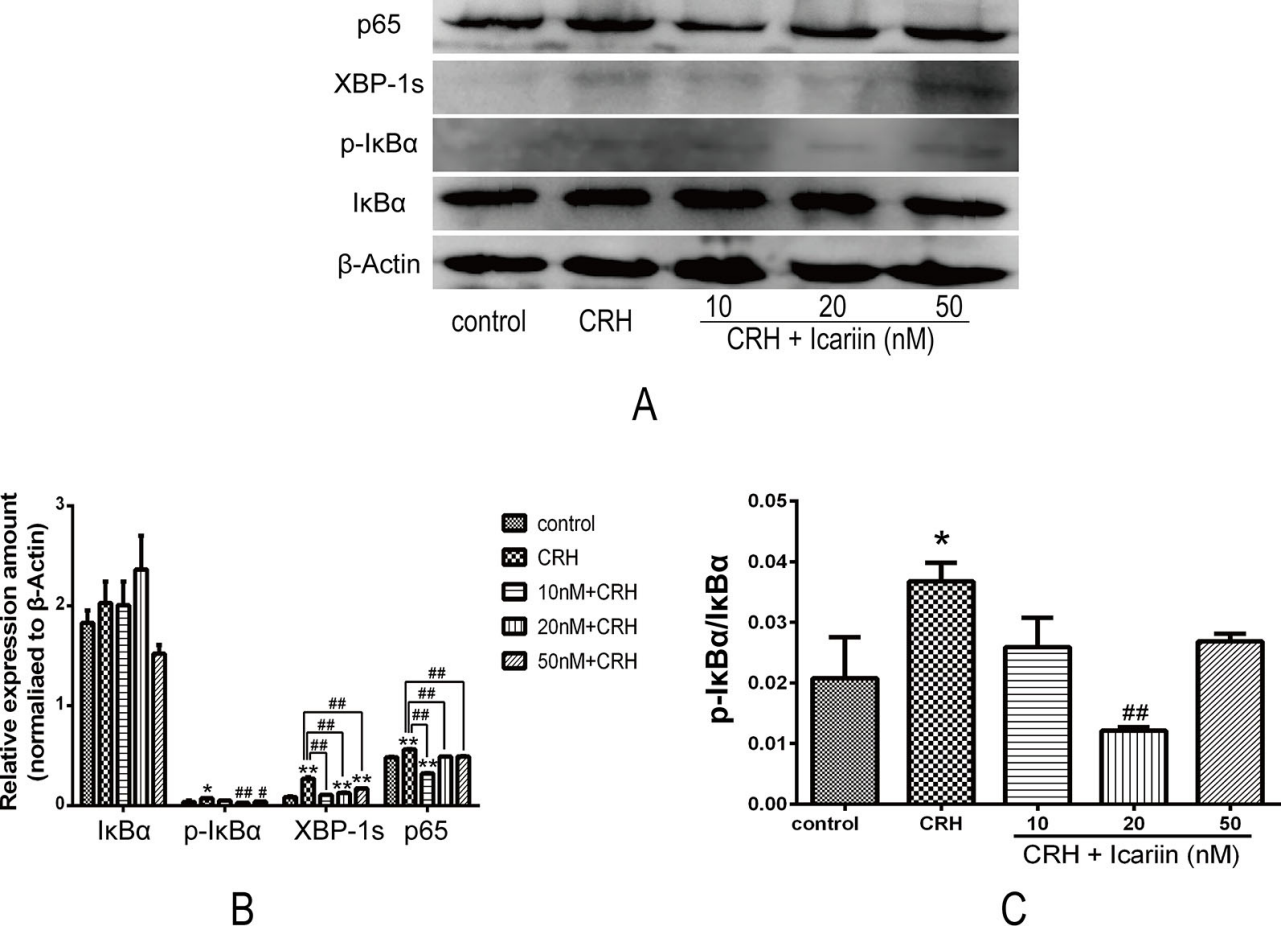
Icariin suppressed expression of XPB-1s and activation of NF-κB pathway in fetal hippocampal neurons. Primary cultured rat hippocampal neurons were pretreated with different concentrations of icariin (10, 20 or 50nM) for 2 h followed by treatment of CRH for 24 h. The immunoblotting **(A)** and the corresponding histogram **(B)** of XPB-1s and NF-κB as well as pIκBα/IκBα **(C)** showed protein expression in different groups. β-actin was reused from **Figure 3** in the assessment of XPB-1s and NF-κB pathway.*p < 0.05 and **p < 0.01 vs control; ^#^p < 0.05 and ^##^p < 0.01 vs CRH.

It is known that NF-κB is crucial in ER stress signaling. We assessed the effects of icariin on NF-κB signaling in primary cultured rat hippocampal neurons. As shown in **Figure 4**, increased expression of p65, p-IκBα, and IκBα were observed after CRH intervention, and further data indicated that icariin treatment caused weakened p65 and p-IκBα protein expression, but IκBα was not significantly decreased. Rate of p-IκBα/IκBα was significantly suppressed by IC20 but not IC50 might be due to possibility of toxicity at 50 nM. XBP-1s expression also eminently increased by CRH and decreased after icariin treatment (**Figure 4**).

### Icariin Reversed Apoptosis Associated Proteins in CRH-Treated Primary Cultured Rat Hippocampal Neurons

**Figure 5:**
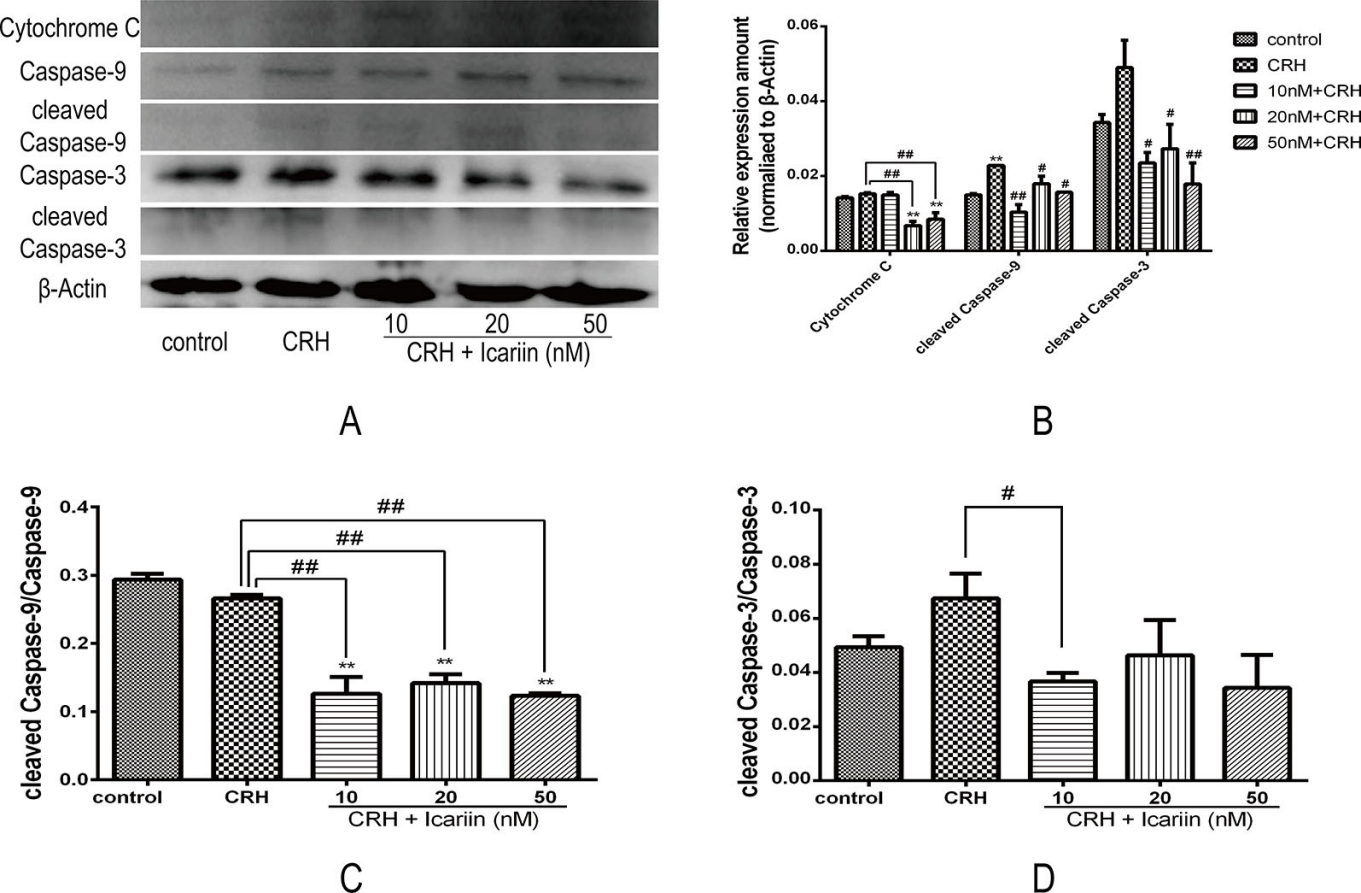
Icariin alleviated apoptosis of hippocampal neurons by reducing expression of apoptosis related proteins in fetal hippocampal neurons. Primary cultured rat hippocampal neurons were pretreated with different concentrations of icariin (10, 20, or 50 nM) for 2 h followed by treatment of CRH for 24 h. The immunoblotting **(A)** and the histogram of apoptosis related proteins **(B)** as well as the ratio of cleaved caspase-9 **(C)** and cleaved caspase-3 **(D)** showed protein expression in fetal hippocampal neurons among different groups. β-actin was reused from **Figure 3**. **p < 0.01 vs. control; *^#^p < 0.05* and *^##^p < 0.01* vs. CRH.

To further monitor the mechanism involved in the neuroprotective effects of icariin under primary cultured rat hippocampal neurons injury, the expressions of apoptosis associated proteins including cytochrome c, caspase-3, and caspase-9 as well as cleaved caspase-3 and cleaved caspase-9 were analyzed by Western blot analysis (**Figure 5**). Distinctly increased level of cleaved caspase-9 was observed and elevating cleaved caspase-3 was also detected in the hippocampal neurons of CRH treatment group, both of which found remarkably decreased after treatments of icariin (**Figure 5**), indicating icariin treatment may play a role in reversing CRH-induced rat hippocampal neurons apoptosis.

Moreover, the level of cytochrome c protein that is related to mitochondrial dysfunction (Eleftheriadis et al., 2016) was also increased compared to that in CRH alone treatment group, and results suggested that icariin could significantly reduce cytochrome c expression in the CRH treated hippocampal neurons (**Figures 5B, D**).

### Icariin Alleviated AHR in Asthmatic Rats

**Figure 6:**
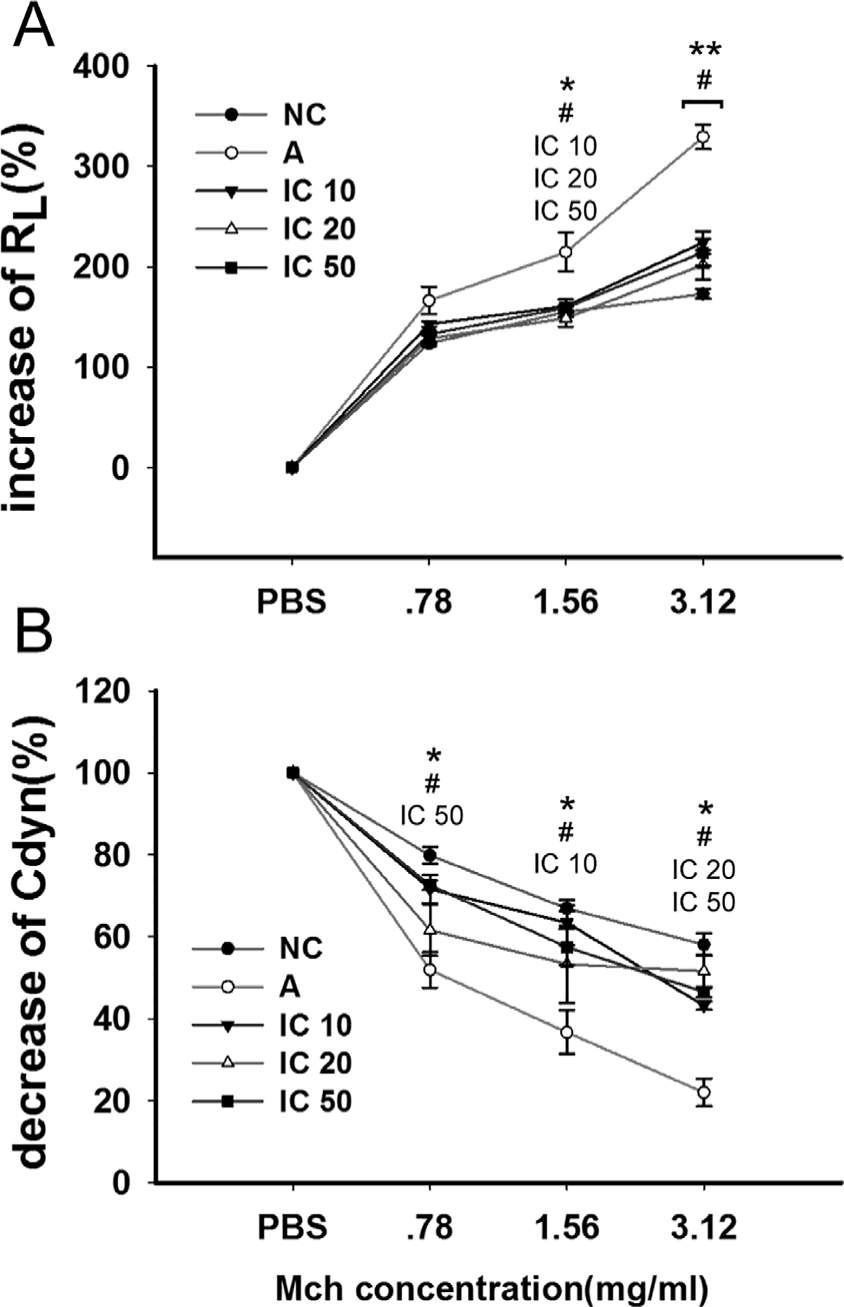
Icariin decreased airway hyperresponsiveness (AHR) to elevating doses of methacholine (Mch) by decreasing airway resistance (RL) and increasing dynamic compliance (Cdyn) in asthmatic rats. The diagram illustrated changes of RL **(A)** and Cdyn **(B)** among different groups. Data were expressed as percentage change from the baseline value. *p < 0.05 and **p < 0.01 vs NC group; ^#^ *p < 0.05* vs A group. IC10, IC25 and IC50 indicated treatments with icariin at 10, 25 and 50 mg/kg, respectively.

The efficacy of icariin on OVA_LPS_-OVA-induced AHR was analyzed by means of the airway responsiveness to aerosolized PBS or Mch. As shown in **Figure 6**, only inconspicuous alterations in R_L_ were observed in normal rats while the OVA_LPS_-OVA-induced asthma model exhibited a significant increase in R_L_ (*p* < 0.05 at 1.56 mg/ml and *p* < 0.01 at 3.12 mg/ml) and decrease in Cdyn (*p* < 0.05 at each dose) compared to the rats in NC group. Icariin of each dose led to an obvious reduction in R_L_ and high dose icariin remarkably promoted Cdyn of asthmatic rats (*p* < 0.05).

### Inflammatory Cell Recruitment in BALF Were Suppressed by Icariin

**Figure 7:**
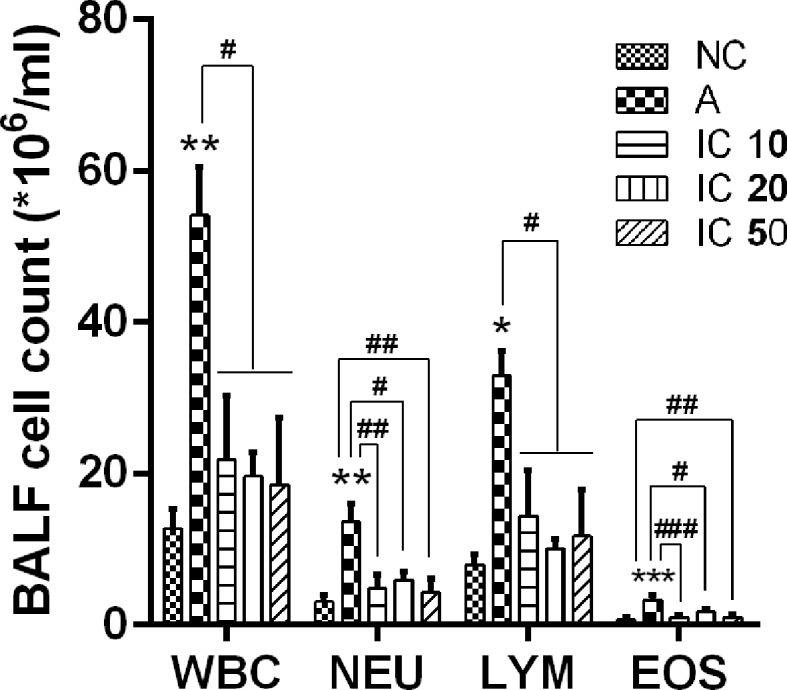
Icariin attenuated influx of inflammatory cells in the lung of asthmatic rats. Total white blood cells (WBC), neutrophils (NEU), lymphocytes (LYM), and eosinophils (EOS) were counted in BALF collected from the lung of rats in each group. *p < 0.05, **p < 0.01 and ***p < 0.001 vs. NC group; ^#^ *p* < 0.05, ^##^ *p* < 0.01 and ^###^ *p* < 0.001 vs. A group.

To ascertain the effect of icariin on the pulmonary inflammatory cells, we detected total white blood cells (WBC), neutrophils (NEU), lymphocytes (LYM), and eosinophils (EOS). LPS-OVA sensitized and OVA challenged rats appeared a large quantity of inflammatory cells influx into the lung (WBC and NEU, *p* < 0.01; LYM, *p* < 0.05; EOS, *p* < 0.001) compared to NC rats (**Figure 7**), Counts of WBC, NEU, LYM, and EOS in BALF were remarkably decreased. Moreover, IC 10 and IC 50 treated rats showed a notably reduction counts in both NEU and EOS (*p* < 0.01 or *p* < 0.001).

### OVA_LPS_-OVA-Induced Lung Histological Changes Were Restored in Icariin-Treated Rats

**Figure 8:**
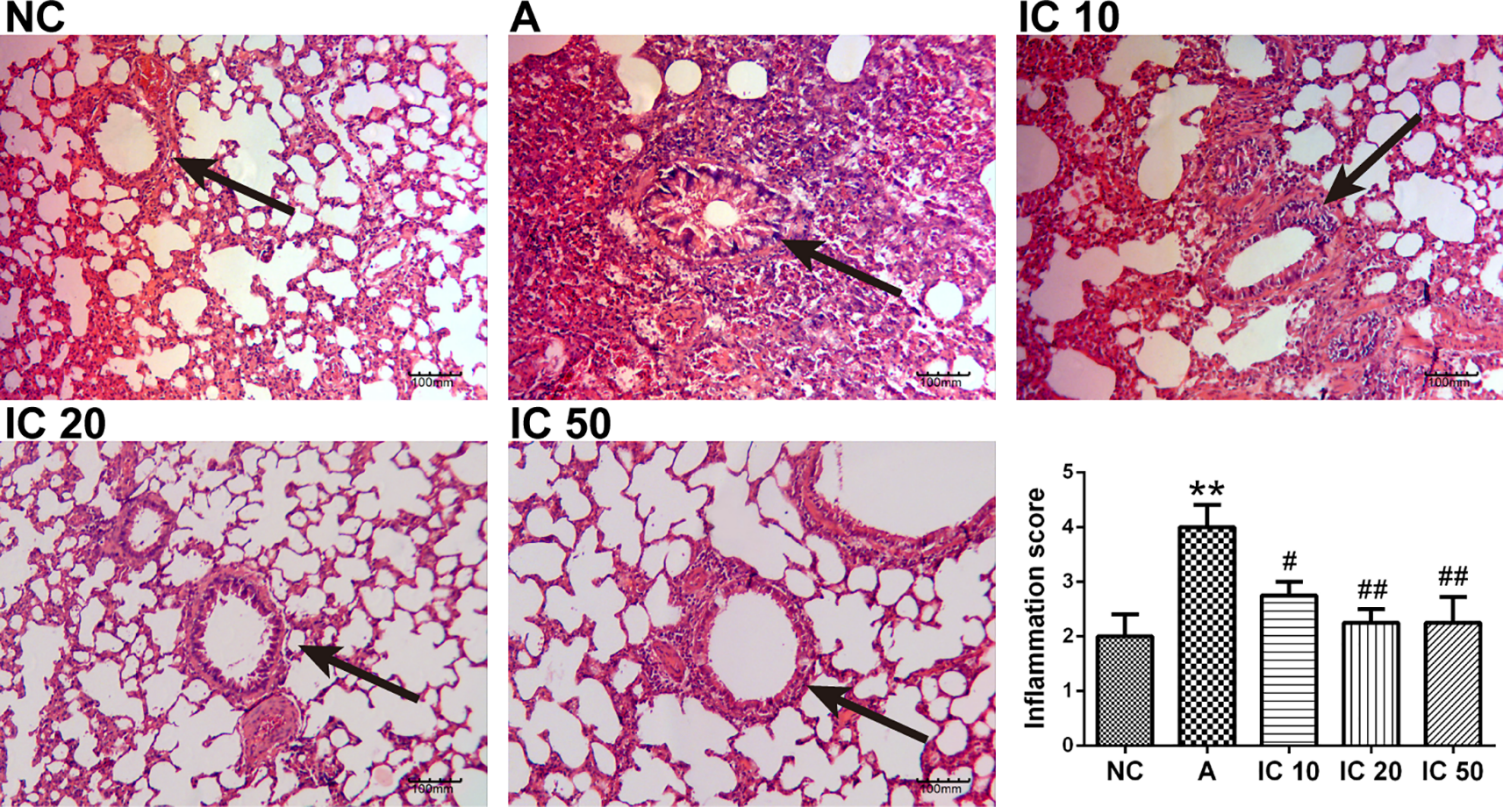
Icariin attenuated inflammation in the lung of asthmatic rats. The histological changes of the lung tissues in each group were investigated by H&E staining. The arrows indicated the changes of inflammation and mucus secretion in each group. Scale = 100 μm (magnification 200×). **p < 0.01 vs. NC group; ^#^ *p* < 0.05 and ^##^ *p* < 0.01 vs. A group.

In OVA_LPS_-OVA exposure asthmatic lung tissues, a distinct infiltration of inflammatory cells and mucus secretion into the tracheobronchial mucosa and airway lumen lesions was observed as compared with the negative control rats (**Figure 8**). In contrast, the icariin treated rats exhibited less infiltration of inflammatory cells into the tracheobronchial mucosa and airway lumen. In addition, IC 20 and IC 50 were more significant (*p* < 0.01, **Figure 8**) in restoring the histological changes than IC 10 did (*p* < 0.05).

### Icariin Decreased IL-1β, IL-6, and TNF-α Level But Increased IFN-γ Level in Serum of Asthmatic Rats

**Figure 9:**
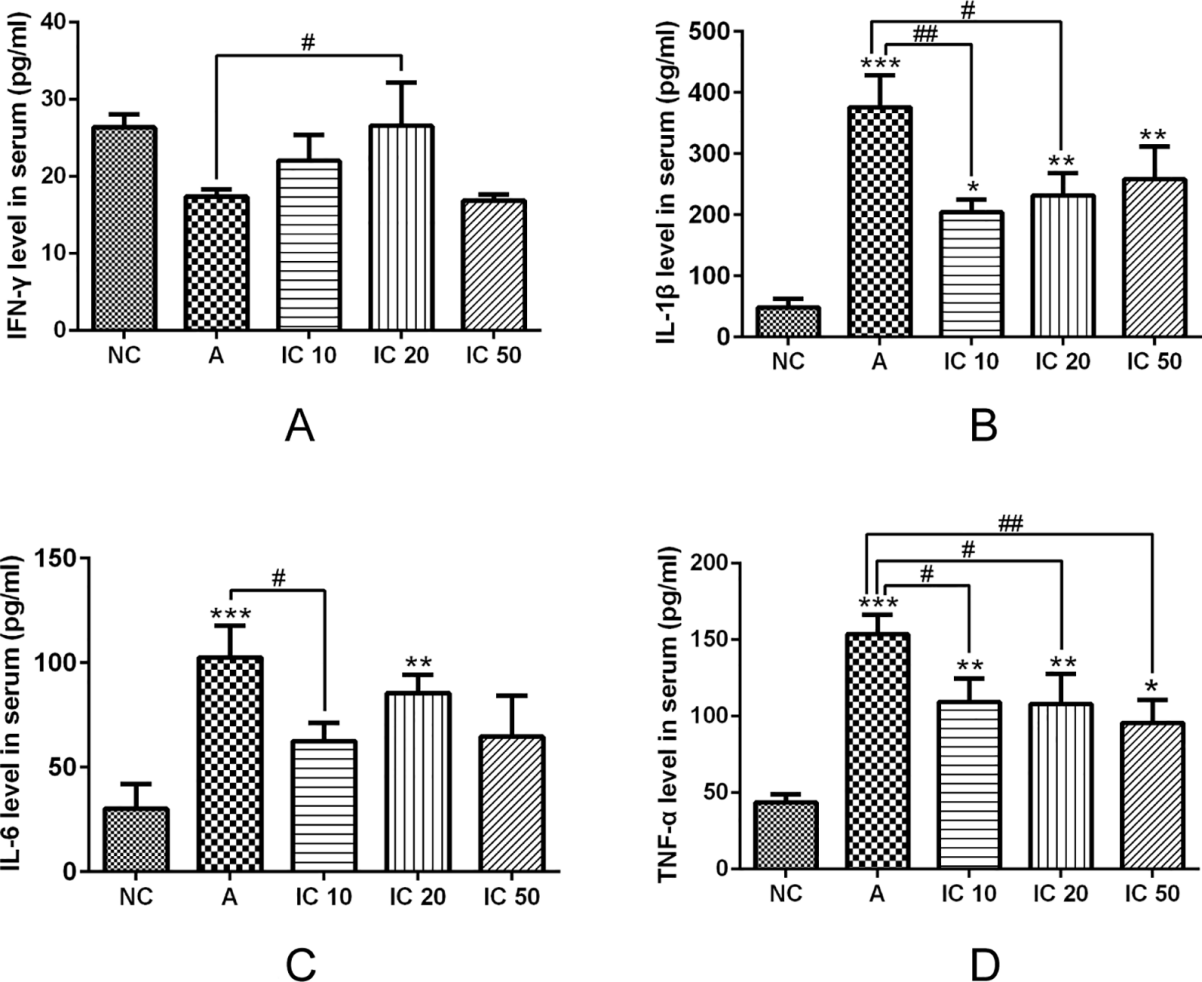
Icariin decreased IL-1β **(B)**, IL-6 **(C)** and TNF-α **(D)** level but increased IFN-γ **(A)** level in serum of asthmatic rats. IFN-γ, IL-1β, IL-6 and TNF-α level in serum were analyzed by ELISA assay. *p < 0.05, **p< 0.01 and ***p < 0.001 vs NC group; *^#^p < 0.05* and ^##^ *p < 0.01* vs A group.

ELISA assay revealed that IL-1β, IL-6, and TNF-α level in serum was significantly increased by OVA_LPS_-OVA stimulation compared to the NC rats (*p* < 0.001, **Figure 9**). Gavage of icariin in each dose showed a reduction in level of IL-1β, IL-6, and TNF-α in serum (**Figures 9B–D**). IL-1β and IL-6 were more significantly reduced by IC 10 (*p* < 0.01 or *p* < 0.05, **Figure 9B, C**). Similarly, TNF-α was more markedly decreased by IC 50 (*p* < 0.01, **Figure 9D**). Conversely, the asthmatic rat models presented a decreasing level of IFN-γ (*p* > 0.05, **Figure 9A**) in serum, but IC 20 treatment evidently increased the level of IFN-γ (*p* < 0.05).

### CRH Was Down-Regulated by Icariin

**Figure 10:**
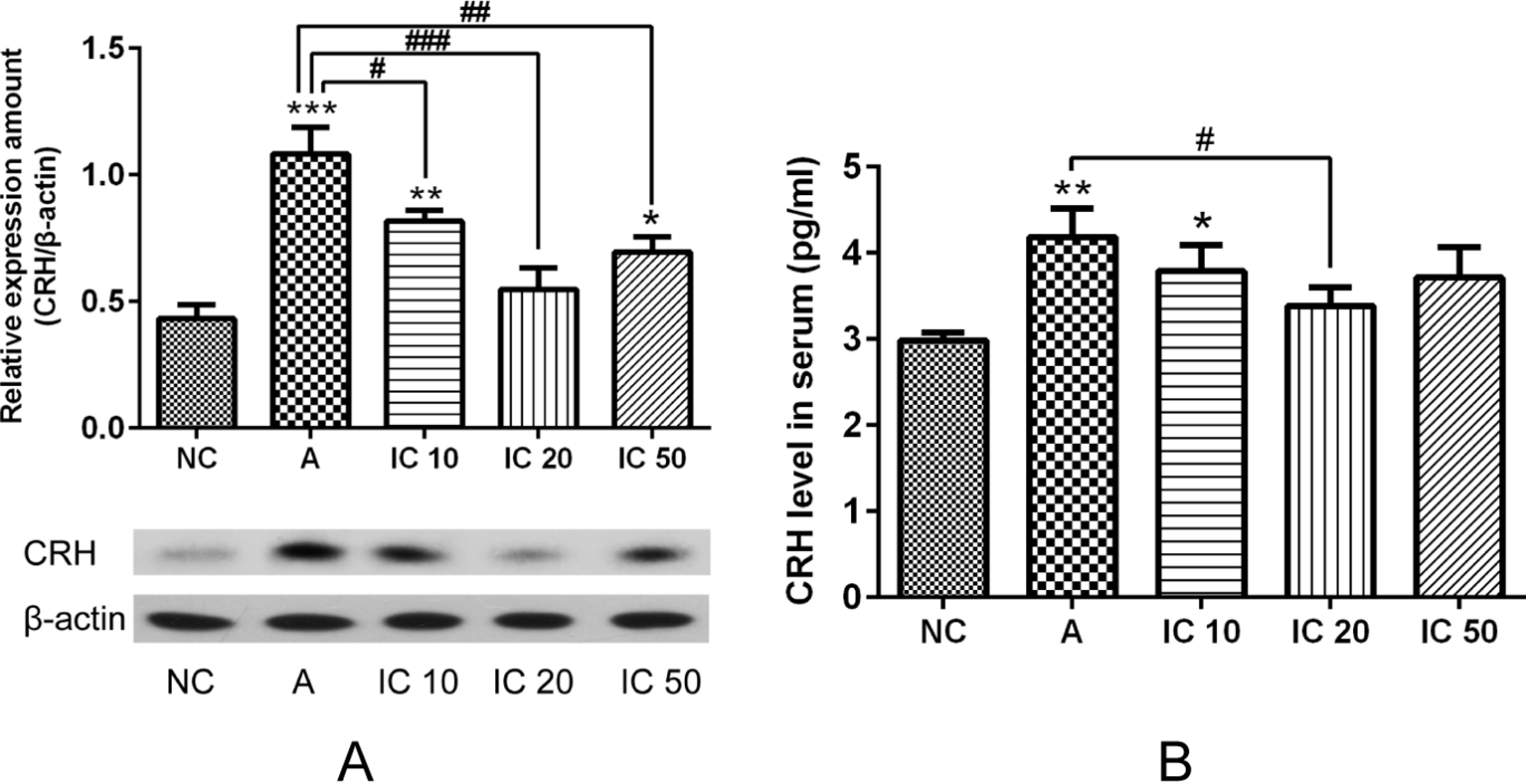
Pretreated with LPS and OVA led to an obvious increment of CRH protein expression in the hippocampus **(A)** and CRH cytokine in serum **(B)**. Icariin at dose of 20 mg/kg exhibited a prominent inhibitory effect both on pulmonary CRH protein expression and serum CRH level. *p < 0.05, **p < 0.01 and ***p < 0.001 vs. NC group; ^#^ *p < 0.05,* *^##^p < 0.01* and *^###^p < 0.001* vs. A group.

As shown in **Figure 10**, pretreated with LPS and OVA led to an obvious increment of CRH protein expression in the lung (*p* < 0.001, **Figure 10A**) and CRH cytokine in serum (*p* < 0.01, **Figure 10B**) of the asthma rats. Interestingly, icariin at dose of 20 mg/kg exhibited a prominent inhibitory effect both on pulmonary CRH protein expression and serum CRH level (*p* < 0.001 or *p* < 0.05).

### Icariin Promoted BiP But Suppressed IRE-1α Protein Expression in the Lung.

**Figure 11:**
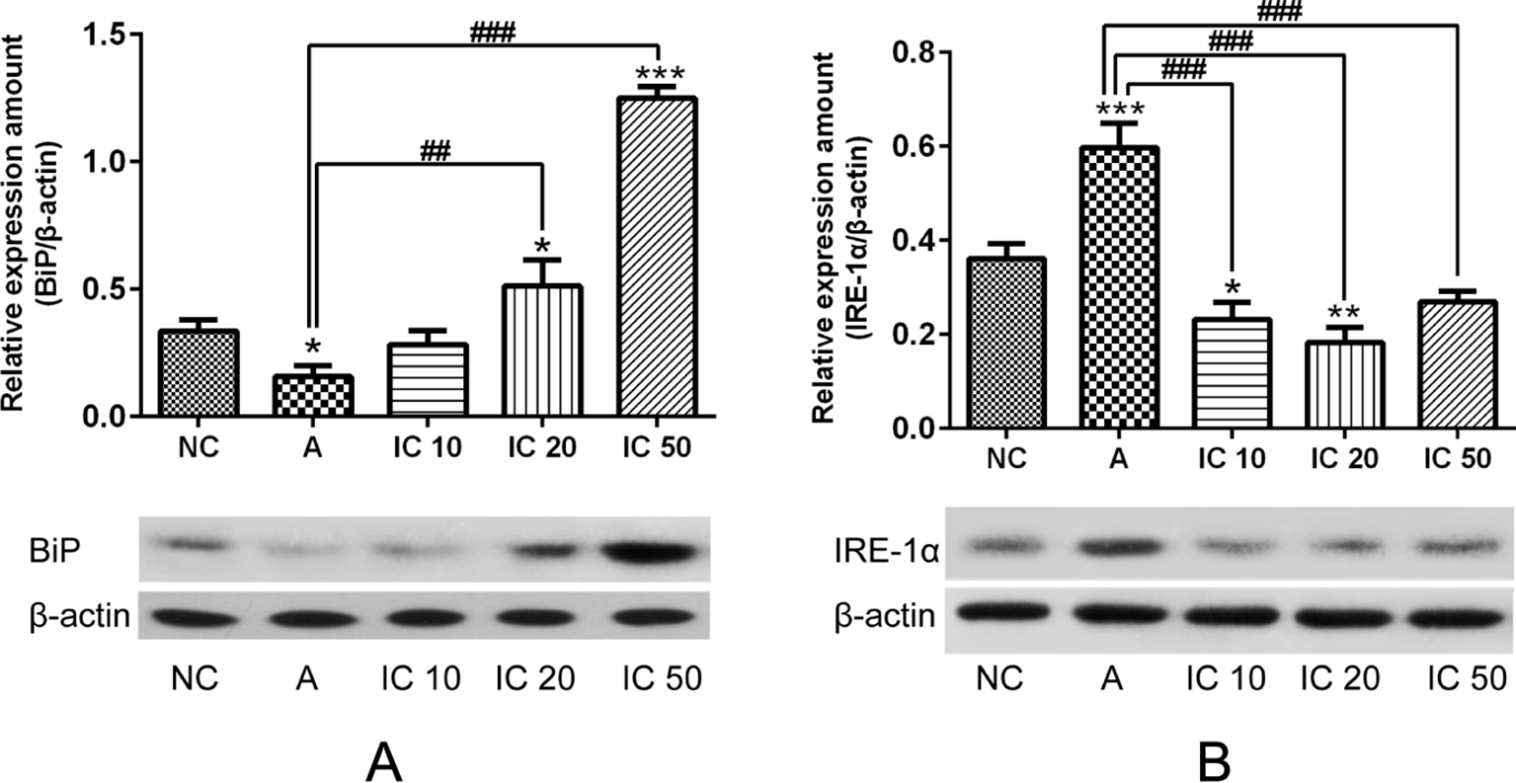
BiP level markedly elevated in a dose-dependent manner in rats pretreated with icariin compared to that in rats treated with CRH alone **(A)**. IRE-1a protein expression in rats was obviously increased by CRH treatment and significantly reduced in the lung of asthmatic rats after each dose of icariin treatment **(B)**. β-actin was reused from **Figure 10** in the measurement of BiP. *p < 0.05, **p < 0.01 and ***p < 0.001 vs. NC group; *^##^p < 0.01* and *^###^p < 0.001* vs. A group.

To further clarify the effect of icariin on BiP and IRE-1α protein expression in the lung of asthmatic rats, we examined the expression of BiP and IRE-1α. The results indicated that BiP level markedly elevated in a dose-dependent manner in rats pretreated with icariin compared to that in rats treated with CRH alone. IRE-1α protein expression in rats was obviously increased by CRH treatment and significantly reduced in the lung of asthmatic rats after each dose of icariin treatment (*p* < 0.001, **Figure 11B**).

### Icariin Decreased XBP-1s Protein Expression and Inhibited NF-κβ in Asthma Rats

**Figure 12:**
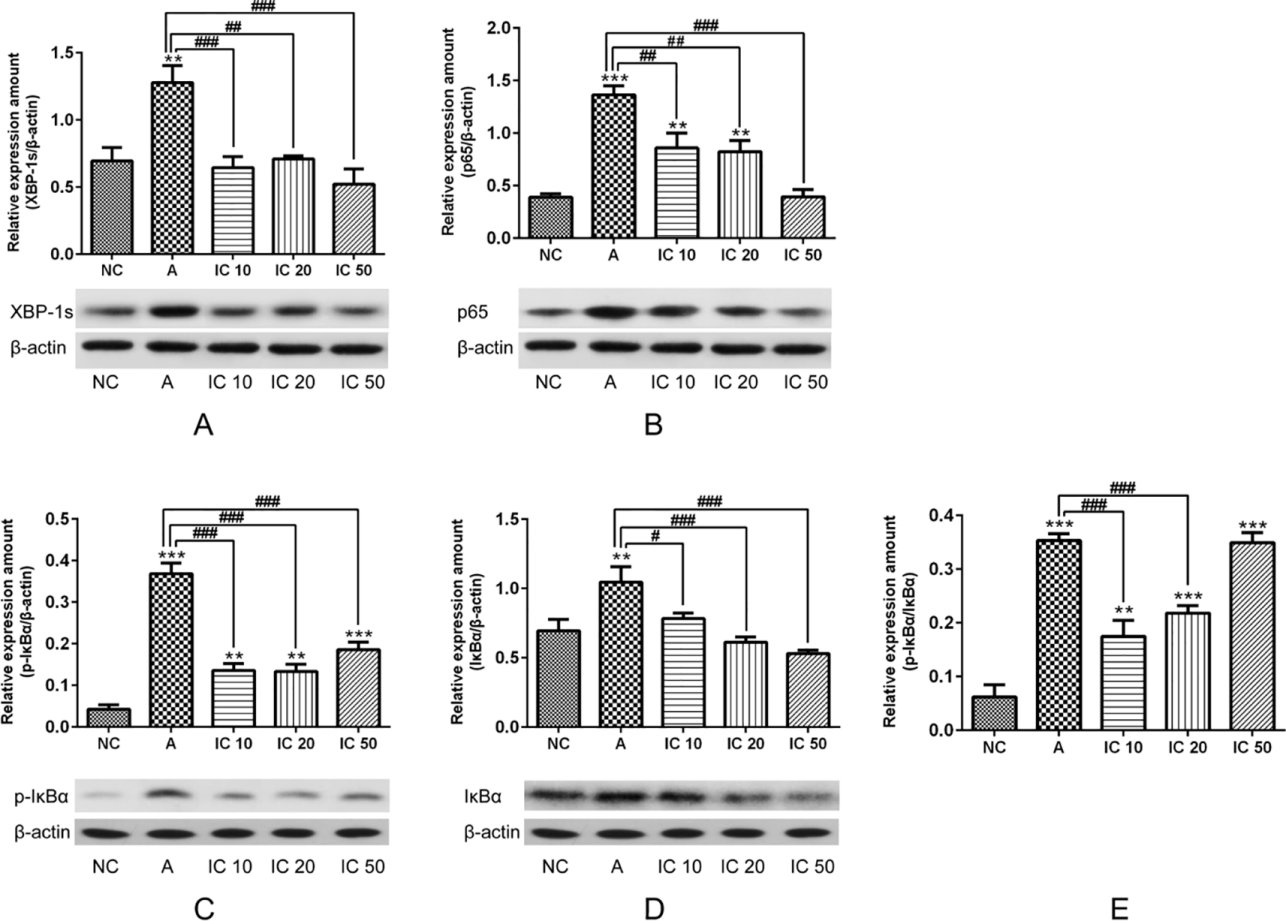
To further demonstrate the efficacy of icariin on XBP-1s and NF-κB, we determined XBP-1s **(A)**, p65 **(B)**, p-IκBα **(C)** and IκBα **(D)** proteins expression as well as ratio of pIκBα/IκBα **(E)**. β-actin was reused from **Figure 11** in the measurement of XPB-1s and NF-κB pathway related proteins. **p < 0.01 and ***p < 0.001 vs NC group; *^#^p < 0.05*, *^##^p < 0.01* and *^###^p < 0.001* vs A group.

To explore the efficacy of icariin on XBP-1s and NF-κB, we further determined XBP-1s protein expression as well as p65, phosphorylated (p-)IκBα, and IκBα proteins expressions. In our results we could find XBP-1s significantly increased (*p* < 0.01, **Figure 12A**) in OVA_LPS_-OVA-induced asthma model and prominently decreased (*p* < 0.001 or *p* < 0.01, **Figure 12A**) after treatment of icariin. Proteins expressions of p65, p-IκBα, and IκBα were observed augmented in asthmatic rats but prominently reduced in icariin treated rats. Moreover, IC 50 exhibited the most effectiveness in blocking p65 and IκBα proteins expressions (*p* < 0.001; **Figures 12B, D**) while IC 20 showed similar effect on p-IκBα (*p* < 0.001; **Figure 12C**).

## Discussion

Asthma is a highly complicated airway inflammatory disorder with its substantially increased incidence. Airway inflammation, AHR, and cytokines production are the major pathological characteristics (Olin and Wechsler, 2014). Mounting evidence had indicated that neutrophils could mediate severe and fatal asthma (Lamblin et al., 1998; Tsokos and Paulsen, 2002; Mauad and Dolhnikoff, 2008), and current asthma treatment guidelines are based predominantly on the T helper type 2 phenotype, and better treatment for severe asthma patients was comprised by the primary unmet needs of current therapies. In this context, we utilized OVA_LPS_-OVA rat to represent as a neutrophilic asthma model. ER stress related signaling networks and NF-κB together were considered to be involved in the pathogenesis and play a critical role in eliciting and maintaining asthma (Kim and Lee, 2015); therefore, the regulation of ER stress and NF-κB could be valuable therapeutic targets and strategies for better control of asthma. Icariin is a major bioactive monomer belonging to flavonoid glycosides attracted from Epimedium, being a classic tonic agent in traditional Chinese medicine. More pharmacological studies indicating that icariin possesses various therapeutic capabilities, especially for neuro-protective (Wang et al., 2017), cardio-protective (Ke et al., 2015), anti-inflammatory, and anti-cancer effects (Fan et al., 2016). In this study, we evaluated the efficacy of icariin on ER stress and NF-κB mediated apoptosis of primary cultured fetal rat hippocampal neurons and OVA_LPS_-OVA-induced asthma rats. The *in vitro* study revealed that CRH-induced ER stress and apoptosis as well as activation of NF-κB were prominently inhibited by icariin treatment in primary cultured fetal rat hippocampal neurons. More importantly, our *in vivo* study also demonstrated that OVA_LPS_-OVA-exposed rats exhibited substantially neuronal ER stress and airway inflammatory changes compared to the normal control groups which were prominently restored by administration of icariin.

CRH is a 41-amino acid peptide with multiple biological effects (Zhang et al., 2012) and produced by the hypothalamus and acts as central modulatory role in response to inflammatory and physiologic forms of stress (Webster et al., 1998). Evidence also suggested that hippocampal dysfunction may be regulated by CRH (Ivy et al., 2010), and intracephalic administration of CRH in immature rats induced apoptosis of hippocampal CA3 neuron independent of glucocorticoids (Brunson et al., 2001). In the present study, CRH was observed dramatically increased in the hippocampus and serum of OVA_LPS_-OVA-induced asthmatic rats. What' s more, the viability of primary cultured fetal rat hippocampal neurons were declined after intervention of 5 nM CRH for 24 h compared to the control group, but pretreatment with icariin significantly promoted the viability of hippocampal neurons indicating icariin has a neuroprotective effect against CRH-induced injury.

Concurrently, we have addressed importance on mechanism underlying immunomodulatory functions of CRH in ER stress, which might be of great value to prevent subsequent asthma. We found CRH was deficient to increase BiP expression while icariin treatment significantly improved the level of BiP both in the fetal hippocampal neurons and the lung tissues of asthma rats in a dose dependent manner. However, the UPR signaling component IRE-1α were markedly augmented in the hippocampus of OVA_LPS_-OVA-induced asthma rats as well as in the primary cultured fetal rat hippocampal neurons after CRH stimulation and remarkably decreased in icariin treatment groups. These data indicates the interaction between ER stress and asthma pathogenic features in rats and potent therapeutic effects of icariin administration.

Moreover, in the current study, we found evidence in primary cultured hippocampal neurons exposed to CRH for a strong relevance between cell apoptosis and ER stress. Cytochrome c, one of the mitochondrial proteins that is released into the cytosol when the cell is activated by an apoptotic stimulus (Ow et al., 2008), was found elevated upon CRH treatment. Our results further found that the cleavage of downstream apoptosis markers caspase-9 and caspase-3 expression were up-regulated in CRH treatment group. However, this upregulation was inhibited by treatment of icariin. Collectively, these data suggested that ER stress participated in the CRH-induced hippocampal neuron apoptosis and could be effectively inhibited by icariin administration.

Inflammatory cytokines were considered to serve as candidates of inflammatory mediators that responsible for the activation of ER stress (Kitamura, 2011). Previous study showed that expression of BiP was induced after *in vivo* injection of IL-6 or IL-1β in mice, and splicing of XBP-1 mRNA was also enhanced upon exposure to these cytokines (Zhang et al., 2006). TNF-α may also induced accumulation of the spliced forms of XBP-1 mRNA and XBP-1 protein and ER stress *via* generation of reactive oxygen species (Xue et al., 2005). Our results showed that IL-6, IL-1β, and TNF-α were dramatically increased in the serum of asthma rats as well as augmented spliced XBP-1 (XBP-1s) both in cultured fetal rat hippocampal neurons and lung tissues of asthma rats, while significantly decreased was then detected with treatment of icariin, which further indicating the suppressive effect of icariin on ER stress in asthma rat models.

NF-κB is a regulatory transcription factor that is important to the expression of inflammatory cytokines in inflammation related diseases (Kuwabara et al., 2012). Study also indicated that NF-κB activation could be triggered by UPR, which expressions of many inflammatory gene were transcriptionally regulated (Tam et al., 2012). Our data indicated that activated ER stress and NF-κB is involved in asthma pathogenesis and administration of icariin remarkably inhibited this activation.

In addition, AHR is another defining feature of asthma, we observed dramatically enhanced AHR in OVA_LPS_-OVA-induced asthmatic rats and demonstrated treatment of icariin led to a marked promotion of Cdyn and reduction of R_L_ in the airway of asthma rats, indicating icariin administration had a therapeutic effect on airway function in asthmatic rats. In addition, pathological changes in the airway consistent with characteristic of asthma were found obviously restored and inflammatory scores markedly decreased by icariin.

In summary, our *in vivo* and *in vitro* studies demonstrated that CRH could induce and mediate ER stress and apoptosis in cultured fetal rat hippocampal neurons and OVA_LPS_-OVA asthma rats, however, this was significantly alleviated by treatment of icariin through inhibiting ER stress related proteins and NF-κB pathway, which would be a valuable therapeutic strategy for stress-induced refractory bronchial asthma.

## Data Availability Statement

The datasets generated for this study are available on request to the corresponding authors.

## Ethics Statement

The animal study was reviewed and approved by the Administrative Committee of Laboratory Animals of Fudan University.

## Author Contributions

JL and LL designed the study. JL, LL, JS, QL, CY, HZ, and FL performed experiments. JL wrote the paper.

## Conflict of Interest

The authors declare that the research was conducted in the absence of any commercial or financial relationships that could be construed as a potential conflict of interest.
