# Supplementary material for: Icariin Protects Hippocampal Neurons From Endoplasmic Reticulum Stress and NF-κB Mediated Apoptosis in Fetal Rat Hippocampal Neurons and Asthma Rats
Source: Front Pharmacol. 2020 Jan 31;10:1660. doi: 10.3389/fphar.2019.01660 (PMC7005524; doi:10.3389/fphar.2019.01660)
Supplement: Supplementary file 1 [file Image_1.pdf]

Supplementary material

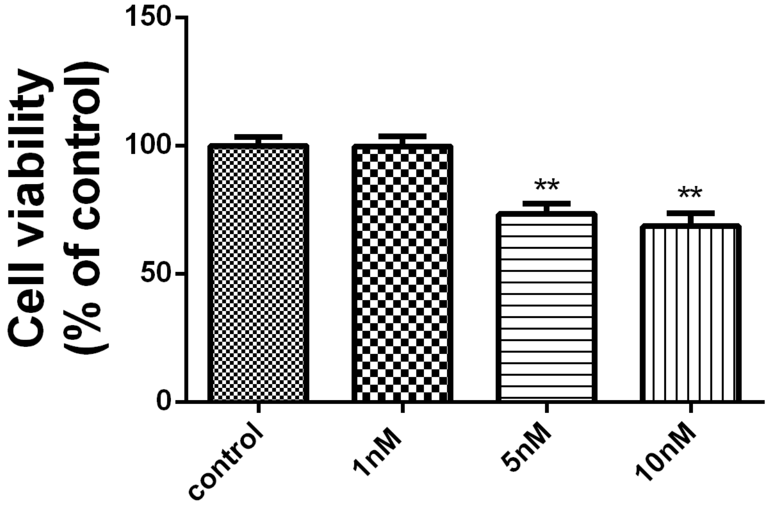

Figure S1. Efficacy of CRH on cell viability of primary cultured hippocampal neurons. Viability of primary cultured hippocampal neurons was detected by MTT 24 h after treatment of CRH with different concentrations (1 nM, 5 nM and 10 nM). ** $p < 0.01$ vs control.
